# Editorial for the Applications and Challenges for Gas Sensors

**DOI:** 10.3390/mi16050493

**Published:** 2025-04-23

**Authors:** Zhaohui Lei, Yinglin Wang, Pengfei Cheng

**Affiliations:** School of Aerospace Science and Technology, Xidian University, Xi’an 710126, China; lzhcatgod@163.com (Z.L.); wangyl@xidian.edu.cn (Y.W.)

Gas sensors, widely used in various fields, are devices used to detect the presence of a specific gas within a certain area or to continuously measure the concentration of gas components [1,2,3,4,5]. The development of gas sensors is well established, but the performance of gas sensors in practical applications is greatly affected by the actual application environment. In the medical field [6], gas sensors can help doctors make rapid diagnoses. In public areas [7], gas sensors can ensure complete safety and security. In environmental monitoring [8], sustainable development can be ensured to the greatest extent. At the same time, the current condition of food can be monitored in real time to guarantee food safety [9].

To better enable the application of gas sensors across diverse fields, the sensitivity, selectivity, and stability of gas sensors need to be improved [10,11]. This topic contains 10 original papers on recent advances in gas sensor research. Specifically, the latest advances in gas sensor applications in the medical field (3 papers), public safety (2 papers), environmental monitoring (3 papers) and food safety (2 papers) are presented. A brief summary is as follows:

1. Medical field

In the medical field, detecting biomarkers in human exhaled breath (such as acetone and ammonia) can facilitate rapid and accurate clinical diagnosis [12]. Humidity poses significant challenges to the gas sensors used in this process. High humidity levels lead to baseline drift and reduced sensitivity in gas sensors [13]. Additionally, exhaled breath analysis necessitates the detection of biomarkers at low concentrations, which demands a low detection limit for gas sensors. Addressing these challenges is critical for the effective application of gas sensors in medical diagnostics.

Lei et al. [14] achieved high performance in the detection of acetone by controlling the structure of a spinel to adjust the concentration of the oxygen vacancy. The performance of the gas sensor under high humidity was improved by introducing rare-earth elements, and the response of the gas sensor under high humidity could still reach 85% of the original response without any significant change in resistance. A simulated human breath was detected using the sensor, and it was found that the sensor can detect acetone at ppb level, which has a great potential for breath detection in diabetic patients.

Li et al. [15] synthesized a novel nanocomposite material for the extremely sensitive detection of NH_3_ in the breath of patients with kidney disease at room temperature. The sensor demonstrated a high selectivity (875%) for 30 ppm NH_3_ and an ultra-low detection limit of 3.7 ppb. The sensor showed good linearity, repeatability and long-term stability. In addition, it could effectively distinguish patients with different stages of kidney disease through quantitative NH_3_ measurements. Through the analysis of the changes in X-ray photoelectron spectroscopy (XPS) signal, the sensing mechanism is clarified, and the adsorption and oxidation pathways of NH_3_ and their effects on charge transfer are explained by density functional theory. The sensor provides a new strategy for the early diagnosis and management of kidney disease.

Li et al. [16] have developed a low-cost, scalable nitrogen oxide (NO_x_) gas sensor. The sensor is based on the design of a moisture-resistant, stretchable NO_x_ gas sensor based on laser-induced graphene (LIG). The LIG sensing and electrode layer, sandwiched between a soft elastic substrate and a moisture-proof semi-permeable encapsulator, is optimized by adjusting laser-processing parameters such as power, image density, and defocusing distance. The gas sensor uses needle-like LIG prepared with optimal laser-processing parameters, has a high response to NO and NO_2_, and has ultra-low detection limits for NO and NO_2_ (8.3 ppb and 4.0 ppb), as well as a fast response/recovery speed and good selectivity. The design of the stretchable snake structure in the LIG electrode and the strain isolation from the rigid island allow the gas sensor to stretch by 30%. Combined with moisture resistance of 90% relative humidity, the gas sensor has been further demonstrated to effectively monitor an individual’s local environment at different times of the day and analyze human respiratory samples to classify patients with respiratory diseases from healthy volunteers. Moisture-resistant, stretchable nitrogen oxide gas sensors could extend the capabilities of wearable devices to detect biomarkers from humans and exposed environments for early disease diagnosis.

2. Public safety

In terms of public safety, the biggest challenge for gas sensors is specificity in detection [17]. With so many similar gases of the same type, how to quickly and efficiently distinguish between them becomes the primary challenge. The improvement of specificity can greatly enhance the accuracy of detection.

Luo et al. [18] discussed the key safety challenges in the field of electric vehicles, with a particular focus on the thermal runaway and gas emissions of lithium-ion batteries. A non-dispersive infrared (NDIR) gas sensor is provided, aiming to effectively monitor battery emissions. Carbon dioxide (CO_2_) gas sensors can enhance the early warning system, facilitate the timely detection of potential problems, and thereby improve the overall safety standards of electric vehicles. A new type of CO_2_ gas sensor based on advanced pyroelectric single-crystal lead niobium magnesium titanate (PMNT) is introduced. Compared with the commercially available materials, it has exceptionally high pyroelectric performance. The specific detection rate of the PMNT single-crystal pyroelectric infrared detector is more than four times higher than that of the lithium tantalate single-crystal infrared detector. This study utilized advanced pyroelectric single-crystal gas sensors to conduct an in-depth exploration of the real-time monitoring of power battery safety. This study also put forward practical suggestions for reducing the risk of thermal runaway in lithium-ion batteries, with particular emphasis on the development of an effective early warning system.

Wu et al. [19] studied the reasons affecting the performance of O_3_ gas sensors through DFT. A strong charge exchange was observed between O_3_ and the β-SnSe monolayer, indicating the existence of chemical adsorption. The calculation of recovery time also indicates that the β-SnSe monolayer can be reused after O_3_ adsorption. The sensitivity of β-SnSe monolayer films to O_3_ was quantitatively evaluated through current-voltage characteristic simulation. The results showed that at a bias voltage of 1.2 V, the sensitivity of β-SnSe monolayer films was as high as 1817.57%. This sensitivity exceeds that of other two-dimensional materials, such as graphene oxide. This comprehensive study indicates that β-SnSe monolayer films have extraordinary potential as a highly sensitive, recyclable, and environmentally friendly O_3_ sensing material.

3. Environmental monitoring

In environmental monitoring, sensors assist in determining air quality by detecting gases that are more harmful to environmental quality, such as NO_2_, H_2_S, and other gases. The diversity and variability of the environment make detection difficult [20]. The main consideration of researchers concerns how to improve the anti-interference ability of gas sensors.

Li et al. successfully obtained hydroxylated graphene oxide by changing the functional groups of the material [21]. While improving the sensitivity of the sensor, the problems of poor response, slow response, and poor anti-interference ability of the sensor were effectively solved. The response of the sensor to other possible indoor gases such as acetaldehyde, ethanol, formaldehyde, n-butanol, etc., was tested. It was found that the sensor has higher selectivity to acetaldehyde, which can better exclude the influence of other interfering gases in indoor environmental detection.

Feng et al. [22] produced a dual-mode PAS (DM-PAS) gas sensor for simultaneous NO_x_ detection. PA signals were generated by modulating LED and QCL light sources in double resonant modes, which were detected by MEMS microphone arrays. The finite-element method was used to optimize the design of DM-PAC with signal-level equalization. Allan deviation analysis showed that the minimum detection limit (MDL) of nitrogen dioxide (NO_2_) and nitric oxide (NO) was 61.5 ppb and 2.0 ppb over the integration time of 450 s. It achieves an accurate detection of similar nitrogen oxides and has great potential in environmental detection.

Li et al. [23] took a ZnCo_2_O_4_ sensor as the research object to explore the influence of small ambient temperature changes on the gas-detection process. The performance of the metal oxide gas sensor is affected by the change in ambient temperature. The significant effect of temperature change on the ZnCo_2_O_4_ sensor is due to the high sensitivity to temperature change, the empty nanocage structure, and the low reactivity of toluene. By constructing regression models of response rate, steady-state resistance, and environmental variables, the influence of ambient temperature on sensing performance was analyzed, which was convenient for the identification of ambient temperature and target gas concentration. This study realizes the accurate identification of toluene concentration using a gas sensor and has a certain guiding effect on environmental detection.

4. Food safety

In terms of food safety, by detecting the unique gases emitted by food volatilization, the state of the food can be quickly and accurately determined [24]. The sensor needs to be more accurate in determining the concentration of specific gases, and it is also necessary that the sensor is capable of more accurate recognition of specific gases at low concentrations.

Xu et al. prepared gas-sensitive materials with dual mesoporous structures and tunable oxygen vacancies by direct solution precursor plasma spraying [25]. A one-step process combining mesoporous structures, precise heterojunction modulation, and controllable oxygen vacancy modulation has been realized in the modified work. The gas-sensitive material can be used for the detection of ethyl undecanone, a key biomarker of rice aging, and exhibits excellent performance at room temperature. The sensor can also be used as an electronic-nose monitoring unit for the rapid identification of adulteration in rice quality. Accurate and timely detection of rice quality was realized.

Lv et al. [26] improved the sensitivity of the sensor to ammonia by adjusting the end functional groups to provide additional ionophores and enhance the carrier transport path. The detection of ammonia at room temperature is realized with good repeatability and selectivity. At the same time, a portable wireless detection device equipped with Bluetooth communication and a microcontroller system was designed to detect the rotting process of fresh shrimp, and wireless real-time monitoring of food freshness was realized through smartphones. This innovative sensing technology has broad application prospects for food-quality monitoring.
